# Chimeric antigen receptor-T cells targeting epithelial cell adhesion molecule antigens are effective in the treatment of colorectal cancer

**DOI:** 10.1186/s12876-024-03286-9

**Published:** 2024-08-06

**Authors:** Siheng Zeng, Ning Jin, Baofeng Yu, Qing Ren, Zhiqiang Yan, Songtao Fu

**Affiliations:** 1 Qingpu Branch of Zhongshan Hospital Affiliated to Fudan University, Shanghai, 201700 China; 2 Birth Defects and Regenerative Medicine Laboratory, Department of Biochemistry & Molecular Biology, Biomedicine and Health Graduate Education Innovation Center, Shanxi Medical University, No. 56, Xinjian South Road, Taiyuan, Shanxi 030001 China; 3 Department of Gynecology, Hainan West Central Hospital, Danzhou, Hainan 571700 China; 4 Biomedicine and Health Graduate Education Innovation Center, Taiyuan, Shanxi 030001 China

**Keywords:** Chimeric antigen receptor T cells, Epithelial-specific adhesion molecules, Colorectal cancer, Cellular immunotherapy

## Abstract

**Objective:**

To construct chimeric antigen receptor (CAR)-T cells targeting epithelial cell adhesion molecule (EpCAM) antigen (anti-EpCAM-CAR-T).

**Methods:**

A third-generation CAR-T cell construct used a single-chain variable fragment derived from monoclonal antibody against human EpCAM. Peripheral blood mononuclear cells were extracted from volunteers. The proportion of cluster of differentiation 8 positive (CD8+) and CD4 + T cells was measured using flow cytometry. Western blot was used to detect the expression of EpCAM-CAR. The killing efficiency was detected using the MTT assay and transwell assay, and the secretion of killer cytokines tumour necrosis factor-α (TNF-α) and interferon-γ (IFN-γ) was detected using the ELISA. The inhibitory effect of EpCAM-CAR-T on colorectal cancer in vivo was detected using xenografts.

**Results:**

It was found that T cells expanded greatly, and the proportion of CD3+, CD8 + and CD4 + T cells was more than 60%. Furthermore, EpCAM-CAR-T cells had a higher tumour inhibition rate in the EpCAM expression positive group than in the negative group (*P* < 0.05). The secretion of killer cytokines TNF-α and IFN-γ in the EpCAM expression positive cell group was higher than that in the negative group (*P* < 0.05). In the experimental group treated with EpCAM-CAR-T cells, the survival rate of nude mice was higher (*P* < 0.05), and the tumour was smaller than that in the blank and control groups (*P* < 0.05). The secretion of serum killer cytokines TNF-α and IFN-γ in tumour-bearing nude mice in the experimental group treated with EpCAM-CAR-T cells was higher than that in the blank and control groups (*P* < 0.05).

**Conclusion:**

This study successfully constructed EpCAM-CAR cells and found that they can target and recognise EpCAM-positive tumour cells, secrete killer cytokines TNF-α and IFN-γ and better inhibit the growth and metastasis of colorectal cancer in vitro and in vivo than unmodified T cells.

**Supplementary Information:**

The online version contains supplementary material available at 10.1186/s12876-024-03286-9.

## Introduction

Colorectal cancer is one of the most common malignant tumours of the digestive system [1], and its therapeutic effect is poor [2, 3]. The most used treatment for colorectal cancer in the early stage is surgical resection of the tumour combined with adjuvant therapies [4–6]. However, patients in the advanced stage have a lower survival rate and a poorer quality of life [7]. Immune cell therapy has garnered significant attention since its inception, especially after achieving impressive results in the clinical treatment of hematologic tumours [8]. The efficacy of immunotherapy technology on solid tumours has also become the direction of focused research.

Immune cell therapy was developed to address the tumour immune escape mechanism characterised by impaired immune recognition, diminished or absent T cell responses and reduced tumour antigen expression [8]. When these immune cells recognise antigens on the tumour surface, they can be activated, release related cytokines and have a cytotoxic impact [9, 10]. The specific implementation involves expressing the single-chain variable fragment (scFv) derived from a tumour-specific antigen antibody on the surface of immune cells through genetic modification. This modification incorporates activation signal sequences in the intracellular region, leading to the up-regulation of related cytokine genes [9, 10]. At present, the application of relatively mature immune cell therapy is chimeric antigen receptor T cell (CAR-T) technology. T cell receptor (TCR)-engineered T cells, TCR CARs and TCR-like CARs can target intracellular neoantigens. These neoantigens are expressed exclusively on tumour cells and not on normal cells. T cell therapy holds an advantage over antibody-based therapies [11]. By engineering T cells to express specific T cell receptors (TCRs) or chimeric antigen receptors (CARs), T cells can more accurately recognize specific antigens on the surface of tumor cells. T cell therapy is able to form long-term immune memory, and this memory response helps to prevent tumor recurrence. Tumor cells are highly heterogeneous, and they can express a variety of different antigens and can even change their surface antigens to evade attack by the immune system. T cell therapy is better able to cope with this heterogeneity due to its high adaptability and diversity, while antibody therapy may be limited by the specific antigen it targets. Therefore, T cell therapy has better efficacy and lower resistance response than antibody-based therapies [11]. Third-generation CAR-T technology research has matured, and its application has achieved great results.

Epithelial-specific adhesion molecule (EpCAM) is a 40 kD type I transmembrane glycoprotein [12], functioning not only as a cancer stem cell antigen but also as a specific antigen for colorectal cancer. The expression of EpCAM antigen is associated with the proliferation and metastasis of tumour cells [13, 14]. Reports have shown that EpCAM antigen expression shows a significant upward gradient in normal cells, dysplastic epithelium and tumour tissues [15]. Although it is also expressed in normal cells, it is not expressed or has a very low expression rate in vital organs [13]. Furthermore, EpCAM expression is almost 100% in common digestive system tumours, such as gastric cancer and colon cancer [16]. In recent years, EpCAM has been a target for immunotherapy, and monoclonal antibodies against it have been approved in Europe for the treatment of malignant ascites in patients with cancer and positive EpCAM expression [17, 18]. Therefore, EpCAM is safe to use as a targeted antigen for CAR-T.

This study constructed a third-generation EpCAM-CAR targeting colorectal cancer antigen EpCAM using scFv from the EpCAM monoclonal antibody as an extracellular single-chain variable region. This study also explored the cytotoxic impact of anti-EpCAM-CAR-T on tumour cells exhibiting both positive and negative EpCAM antigen expression, both in vitro and in vivo. These investigations establish a fundamental research foundation for the clinical application of CAR-T treatment targeting the EpCAM antigen in colorectal cancer.

## Materials and methods

### Animals

Twenty-four female BALB/C nude mice were bred according to specific pathogen-free feeding standards. The nude mice were purchased from Watson Crick (Beijing) Biotechnology Co., Ltd. Tumour xenografts were performed at the age of 6 weeks. The injected cells were HT29 cells, and the number of injected cells was 2 × 10^6^. The model was constructed using subcutaneous implantation, and the tumour cell suspension was injected into the left lower abdominal region of the nude mice. Tumour formation was evident through observable signs, such as a distended abdomen and palpable masses of varying sizes. These manifestations indicate the successful establishment of a colorectal cancer tumour-bearing nude mouse model.

Following tumour formation in the nude mice, six mice were randomly chosen as a group. Nude mice with comparable tumour sizes and body weights were then selected and evenly divided into four groups, each comprising six mice. The groups were as follows: the blank group (injected medium), the T cell group (injected common T cells), the empty vector group (injected T cells transfected with empty vector) and the CAR-T group (injected T cells transfected with EpCAM-CAR). The cells corresponding to each group were administered at a dose of 2 × 10^7^/kg through the tail vein. Injections were performed on days 7, 14, 21 and 28, with concurrent measurements of tumour volume and documentation of any occurrences of mortality among the nude mice. On the 30th day, cardiac blood sampling was conducted to collect blood from the mice. Mice were anesthetized using isoflurane and dissected following anesthesia. Isoflurane was administered at 2% isoflurane and 100% oxygen [19]. Subsequently, the blood was centrifuged, and the serum obtained was utilised for enzyme-linked immunosorbent assay (ELISA) experiments, aiming to detect the expression of relevant cytotoxic impact factors. The tumours were removed from the nude mice, and the tumour volume (1/2 × long diameter × [short diameter]^2^) and tumour weight were measured, and the death of the nude mice was recorded.

### Cell lines and cell culture

Peripheral blood mononuclear cells were extracted from volunteers. All donors were informed and signed an informed consent form. The study was approved by the hospital ethics committee. Human embryonic kidney 293T (HEK293T), HT-29 (EpCAM+), HCT116 (EpCAM+) and HeLa (EpCAM-) cell lines were purchased from ATCC (Shanghai, China). Moreover, HEK293T cells were cultured in DMEM-High Glucose supplemented with 10% foetal bovine serum (FBS) and 100 mg/mL of penicillin/streptomycin/glutamine) (PSG) (Gibco). Following this, HT-29, HCT116 and HeLa cells were cultured in RPMI-1640 medium supplemented with 10% FBS and 100 mg/mL of PSG (Gibco) before T cells were cultured in GT-T551 supplemented with 10% FBS, interleukin-2 (IL-2)(200 U/ml)and 100 mg/mL of PSG. T cell activation and expansion were performed using cluster of differentiation 3/cluster of differentiation 28 beads (CD3/CD28) and IL-2. This study involved transferring 25 µl of beads (for 1 × 10^6^ cells) into an EP tube and supplementing them with 1 ml of cell culture media. After allowing the magnetic stand to settle for 1 min, the supernatant was discarded. Subsequently, IL-2 was added to the resuspended beads in 1 ml of cell culture medium to achieve a final concentration of 10 ng/ml. The resulting cell pellet, now suspended in the medium containing activated beads and IL-2, was then transferred to a 24-well plate and incubated in the laboratory incubator for 24 h.

### Lentivirus production and infection

The psPAX2 vector and pCMV-VSV-G and a customised Lenti-EF1a-puro plasmid containing the desired gene were co-transfected into HEK293T cells. Following a 24-hour incubation period, the culture medium was refreshed. After 72 h, the medium, now containing lentivirus, was collected. For lentiviral infection, this viral-containing medium was added to the conditioned culture medium. After an additional 48 h, the medium was replaced with a fresh culture medium, and selective drugs were introduced for resistance selection.

### Flow cytometry

T cells infected with EpCAM-CAR lentivirus were observed for green fluorescence under a microscope 3 days later, and by day 6, they were blown out and counted, and the concentration was adjusted to 1 × 10^6^/ml. Three flow tubes were taken and labelled as experimental, control and blank groups. Human recombinant EpCAM antigen was used as the primary antibody and incubated at room temperature for 60 min. Phycoerythrin (PE)-labelled goat anti-human EpCAM flow antibody was added as the secondary antibody to the experimental panel control group and incubated in the dark for 20 min. The expression of EpCAM-CAR on the cell membrane of T cells was detected using flow cytometry.

### Immunohistochemistry

Paraffin wax was used for embedding the sample tissues. Deparaffinisation of paraffin sections (thickness = 5 μm) was performed using xylene and graded alcohols. Antigen retrieval was performed using microwave treatment with a citric acid solution (pH 6.0) for 20 min. Samples were then cooled at room temperature and subsequently treated with 3% hydrogen peroxide for 10 min, also at room temperature, to quench endogenous peroxidase activity. Nonspecific binding was mitigated by incubating with goat serum for 1 h. Subsequently, staining was conducted using haematoxylin-eosin or overnight at 4 °C with the following primary antibody: Ki67 (9449, CST). Staining was examined using HRP Envision Systems (Dako, Shanghai, China).

### Real-time polymerase chain reaction

T cells were transfected with a lentiviral virus, and intracellular RNA was collected by lysing samples with the TRIzol® reagent. Furthermore, cDNA was prepared by reverse transcription (Bio-Rad, Berkeley, CA). FastStart SYBR® Green Master (Roche, Indianapolis, IN) was used to perform quantitative polymerase chain reaction (PCR)-reverse transcription. The reaction mixture underwent incubation at 50 °C for 15 min, followed by a denaturation step at 95 °C for 5 min. Subsequently, 40 PCR cycles were performed with the following temperature profiles: 95 °C for 15 s, 60 °C for 30 s and 72 °C for 1 min. Gene expression values were normalised to those of β-actin, and data processing was performed using the 2^−ΔΔCt^ method.

### Cell coculture assay

This experiment was divided into four groups: the control group, untreated EpCAM negative and positive tumour cells; the T cell group, common T cells co-cultured with EpCAM positive and negative cells; the empty vector group, T cells co-cultured with tumour cells transfected with empty vector lentivirus; and the EpCAM-CAR-T group, T cells co-cultured with tumour cells transfected with EpCAM-CAR-LV5 lentivirus. Each group was further divided into two different effector-to-target ratios of 5:1 and 10:1 for comparison. In the original 96-well plate implanted with tumour cells, after removing the culture medium, 100-µL effector cells of each group were added to each well and cultured in a 37℃-incubator containing 5% CO_2_ for 6–24 h. After centrifugation, the supernatant was aspirated for an ELISA experiment to detect the expression of related cell cytotoxic impact factors.

### Enzyme-linked immunosorbent assay

The ELISA experiment comprised blank wells, standard wells, and the sample wells designated for testing (containing the cell supernatant to be analysed). Each well received 100 µL, slowly instilled into the sample and mixed through gentle shaking. Following the addition of the liquid sample and standard, the microplate was sealed with a liquid using a microplate sealer and incubated at 37℃ for 40 min. Subsequently, a washing solution was applied. Distilled water (50 µL) was added to each well, followed by the addition of 50 µL primary antibody to each well (excluding the blank well). The reaction plate was placed in a 37℃ oven for incubation for 25 min. Next, 100 µL of enzyme-labelled reagent, equilibrated to room temperature, was added to each well (excluding the blank well) and incubated in a 37℃ oven for 15 min. Subsequently, 100 µL of substrate working solution was added to each well, followed by gentle shaking or blowing and incubation in a 37℃ oven for 20 min in the dark. Finally, 100 µL of stop solution was added to each well, with careful attention to a slow drip from the wall, and it was gently blown and beaten evenly. The microplate reader measured the optical density (OD) value of each well at 450 nm, with the blank well serving as the reference point for zero calibration.

### MTT assay

Following a two-day co-culture of T cells with tumour cells, the supernatant underwent centrifugation, leaving the cells intact. Subsequently, each well was treated with 20 µL of MTT for 4 h. After discarding the MTT, 150 µL of DMSO was added and shaken on a shaker for 10 min. The light absorption value of each well was then measured on a microplate reader at a wavelength of 490 nm, using the blank well as a reference for zero calibration. The recorded OD values were saved, and the data were subjected to analysis. To calculate the relative viability of cells, the following formula was applied: cell viability (%) = (OD of the experimental group − OD of the blank group)/(OD of the control group − OD of the blank group) × 100%.

### Transwell assay

T cells were inoculated into transwell inserts with co-cultured tumour cells. Cells in the lower chamber were fixed using 4% paraformaldehyde after 48 h, and they were subsequently stained using a crystal violet solution. The number of migrated cells was recorded using a microscope.

### Statistical analysis

All data were analysed using the Graph Pad Prism 7.0 software. All experimental results were presented as measurement data, expressed as mean ± standard deviation. The differences in cytokines and cytotoxic impact of CAR-T cells were analysed using the *t*-test. Subsequently, survival curves were generated using Excel following the analysis conducted with the SPSS software. A value of *P* < 0.05 was considered statistically significant.

## Results

### Design of epithelial-specific adhesion molecule chimeric antigen receptor

**Fig. 1:**
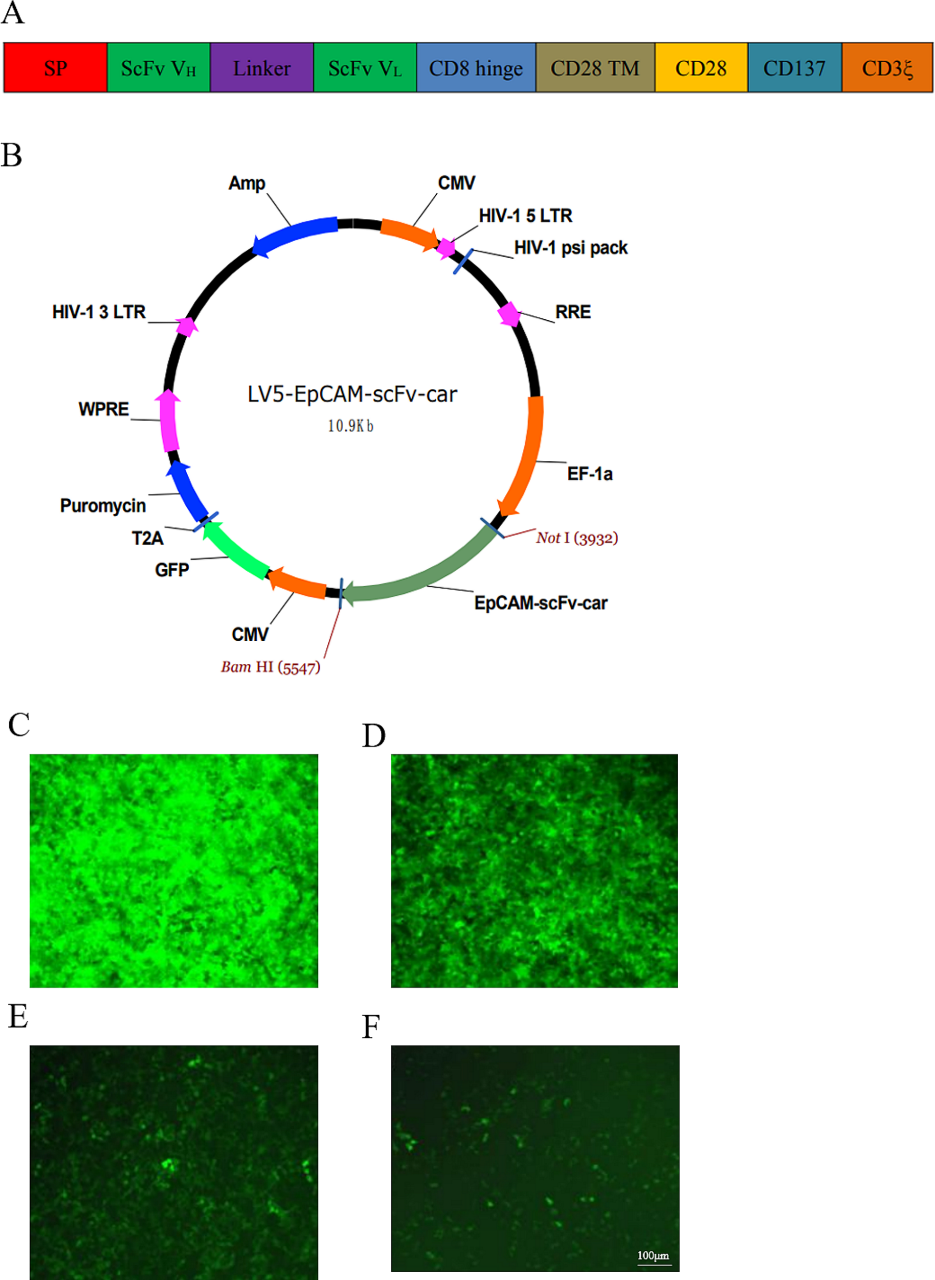
Design process for EpCAM-CAR. **(A)** Structure diagram of EpCAM-CAR. **(B)** Schematic of plasmid construction for EpCAM-CAR. C-F. Lentiviruses were diluted 10^− 1^ fold **(C)**, 10^− 2^ fold **(D)**, 10^−−3^ fold **(E)**, and 10^− 4^ fold **(F)** to infect HEK293T cells

After determining the sequence of each fragment, the third-generation EpCAM-CAR was constructed by connecting the scFv region sequence of the anti-EpCAM antibody with the CD8 hinge region, CD28 transmembrane region, CD28 signal region, CD137 signal region and CD3ξ signal region in turn [20]. The variable heavy (VH) chain and variable light (VL) chain of the scFv were connected using linker sequences. Additionally, the EpCAM-CAR structure was enhanced by incorporating the CD8α signal peptide sequence and Kozak sequence at its forefront, aiming to optimise gene expression and localisation efficiency (Fig. 1A). The sequences shown in Fig. 1A were constructed into the LV5 lentiviral plasmid (Fig. 1B). Subsequently, the coating of lentivirus was performed to determine the lentiviral titer (Fig. 1C-F). The above results indicated the successful construction of EpCAM-CAR.

### Construction of epithelial-specific adhesion molecule chimeric antigen receptor T cells

**Fig. 2:**
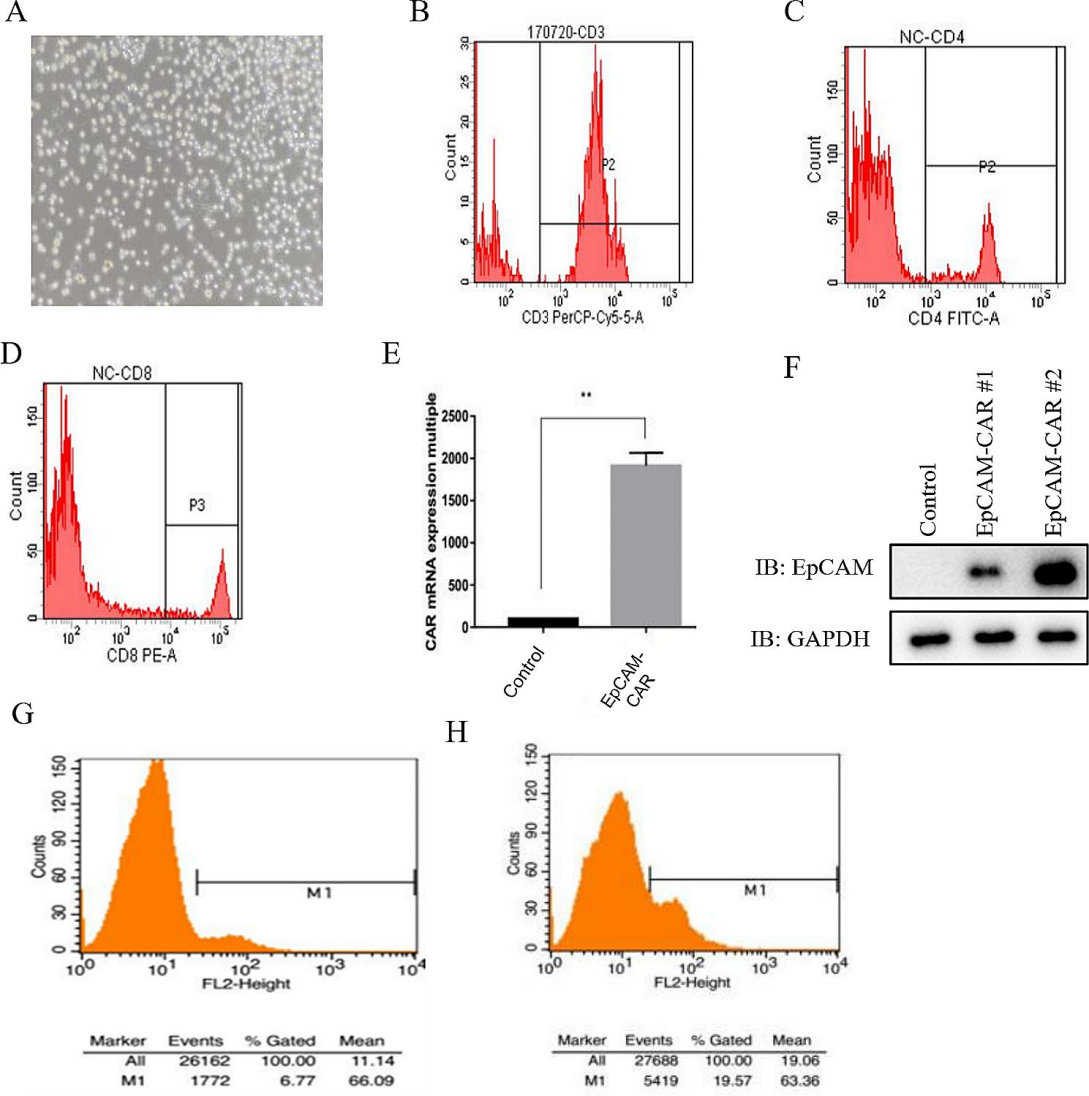
Construction and validation of EpCAM-CAR-T cells. **A** Representative pictures of PBMCs culture. **B**-**D**. CD3+(B), CD4+(C) And CD8+(D) T cells detected by flow cytometry. **E**. Expression of EpCAM-CAR mRNA was detected by qPCR. Error bars mean ± SD, **: *p* < 0.01, by two-tailed unpaired student’s t-test analysis. **F**. Expression of EpCAM-CAR protein was detected by western blot. **G**, **H**. Expression of EpCAM-CAR protein was detected by flow cytometry (G: Control cells, H: EpCAM-CAR-T cells)

Human peripheral lymphocytes were obtained from human blood and successfully cultured for activation (Fig. 2A). Detection of T cells was performed using flow cytometry. The results showed that the proportion of CD3-positive T cells was 25%, the proportion of CD3^+^CD4^+^ T cells was 48%, and the proportion of CD3^+^CD8^+^ T cells was 40% (Fig. 2B-D). T cells were infected with EpCAM-CAR lentivirus, and the EpCAM-CAR expression was detected using qPCR. The results showed that the EpCAM-CAR expression significantly increased in T cells (Fig. 2E). Furthermore, the EpCAM-CAR expression in T cells was subsequently detected using the western blot and flow cytometry methods, which showed that EpCAM-CAR was successfully expressed in T cells (Fig. 2F-H). The above results indicate the successful construction of EpCAM-CAR-T cells.

### Secretion detection of cytokines

**Fig. 3:**
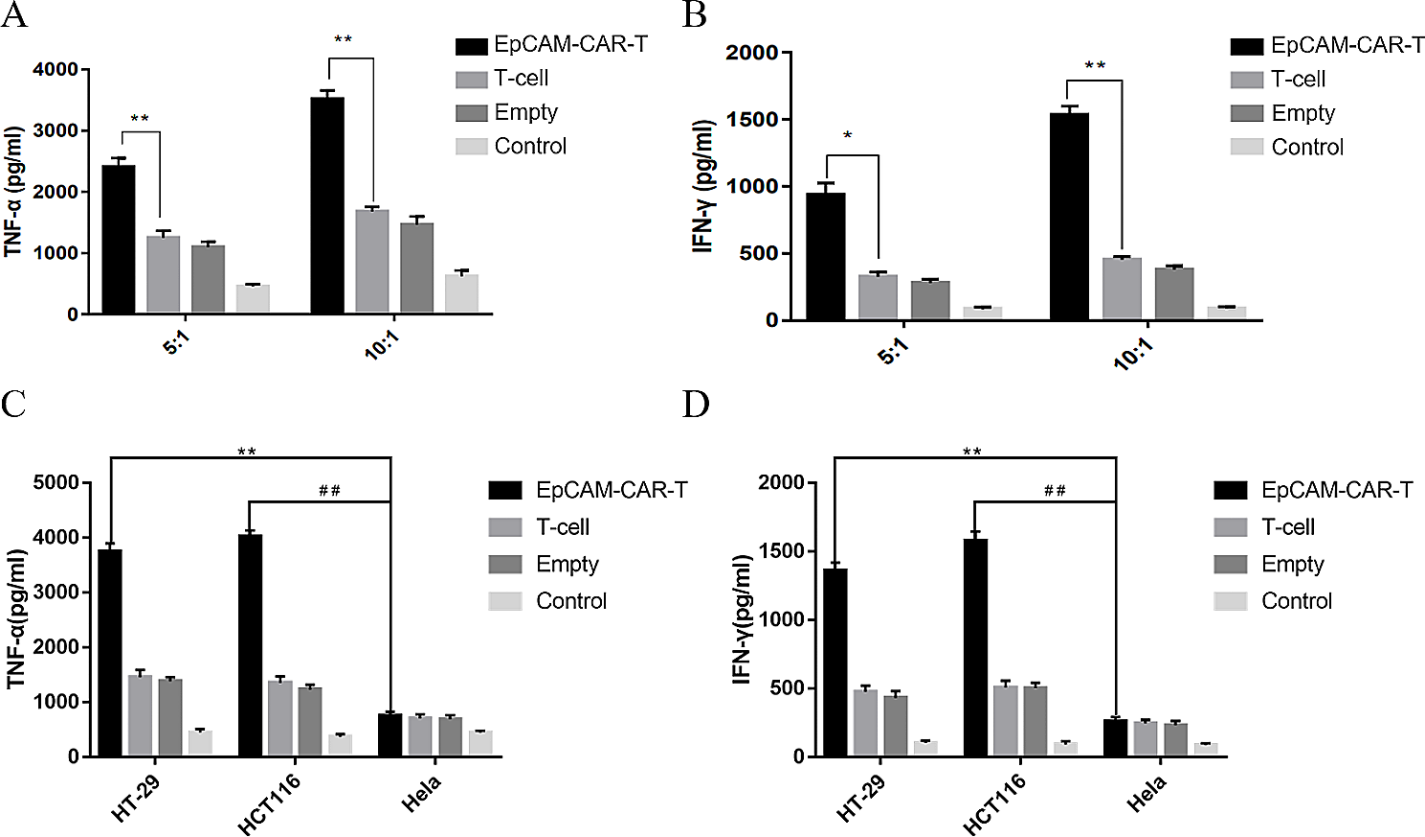
The cytokine secretion of T-cells. **A**: Secretion of TNF-α after coculture of two effector-to-target ratios with HT-29 cells. Error bars mean ± SD, *: *p* < 0.05, **: *p* < 0.01, by two-tailed unpaired student’s t-test analysis. **B**: Secretion of INF-γ after coculture of two effector-to-target ratios with HT-29 cells. Error bars mean ± SD, **: *p* < 0.01, by two-tailed unpaired student’s t-test analysis. **C**. TNF-α secretion in different effector cells co-cultured with HT-29, HCT116 and Hela cells. Error bars mean ± SD, **: *p* < 0.01, ##: *p* < 0.01, by two-tailed unpaired student’s t-test analysis. **D**. INF-γ secretion in different effector cells co-cultured with HT-29, HCT116 and Hela cells. Error bars mean ± SD, **: *p* < 0.01, ##: *p* < 0.01, by two-tailed unpaired student’s t-test analysis

This experiment was divided into the EpCAM-CAR-T group, T-cell group, empty vector group (transfected empty vector lentivirus group) and control group (no effector cell group). Two effector-to-target ratios were set for each group, and both effector-to-target ratios effectively induced TNF-α and INF-γ production (Fig. 3A, B). Co-culture with HT-29 clearly induced TNF-α and INF-γ production compared with co-culture with HeLa (Fig. 3C, D). The above results showed that co-culture with EpCAM-positive cells induced TNF-α and INF-γ production by EpCAM-CAR-T cells.

### Cytotoxic effect of epithelial-specific adhesion molecule chimeric antigen receptor T cells on tumour cells in vitro

**Fig. 4:**
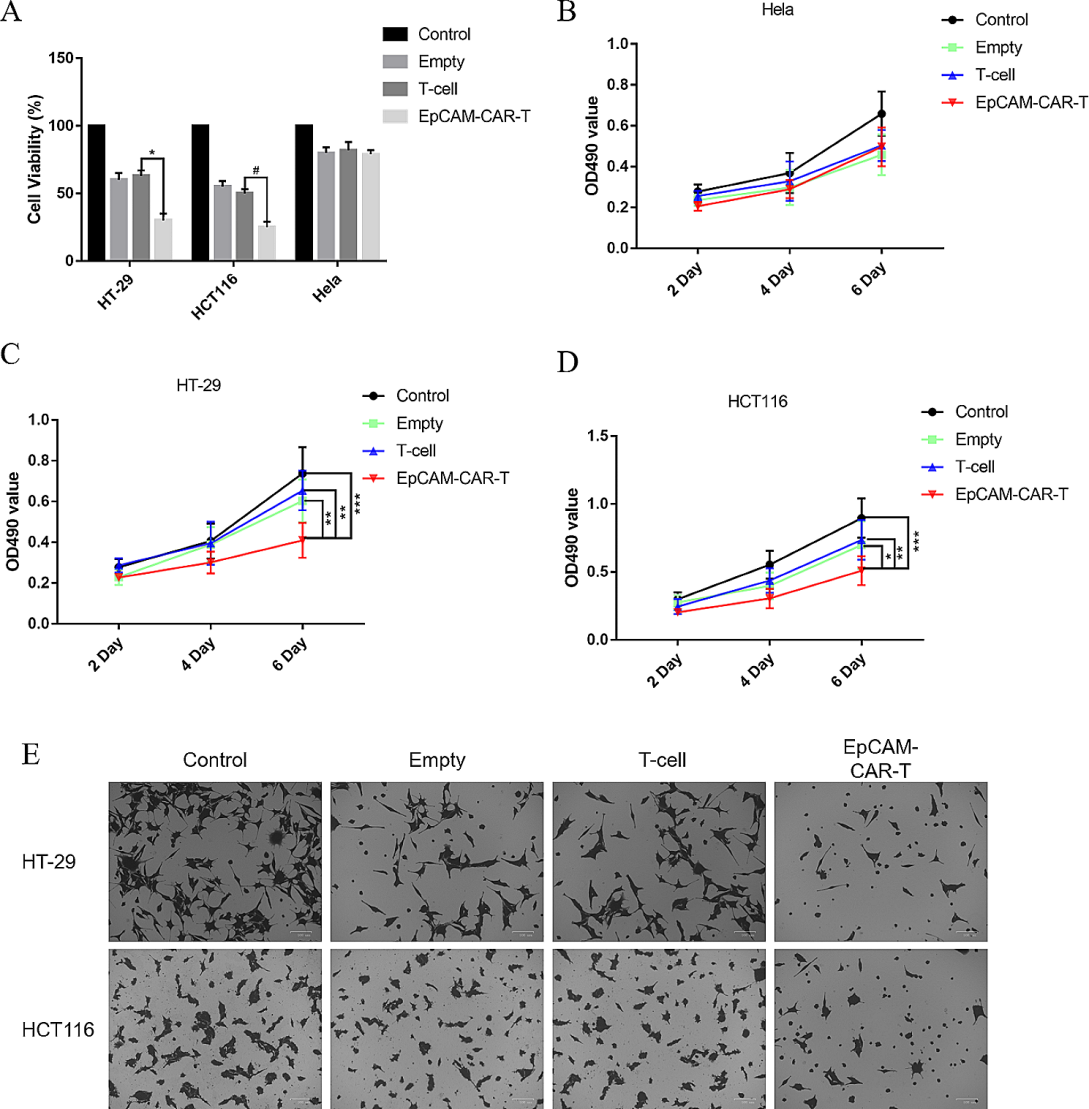
Verification of killing effect of EpCAM-CAR T cells in vitro **A**: Effect of different effector cells on the viability of HT-29, HCT116 and Hela cells. Error bars mean ± SD, *: *p* < 0.05, #: *p* < 0.05, by two-tailed unpaired student’s t-test analysis. **B**-**D**. Effect of different effector cells on growth rate of Hela **(B)**, HT-29(C) and HCT116(D) cells. Error bars mean ± SD, *: *p* < 0.05, **: *p* < 0.01, ***: *p* < 0.001, by two-way ANOVA analysis. **E**: Effect of different effector cells on migration of HT-29 and HCT116 cells

Cell viability was assessed through MTT analysis following the co-culture of effector cells with tumour cells in each experimental group. The results showed that EpCAM-CAR-T cells effectively inhibited the cell viability of HT-29 and HCT116 with positive EpCAM expression (Fig. 4A). The co-culture of EpCAM-CAR T cells with HeLa cells did not exert a significant inhibitory effect on EpCAM-negative HeLa cells (Fig. 4B). Conversely, the co-culture of EpCAM-CAR T cells with HT-29 and HCT116 cells resulted in a notable reduction in the growth rate of both HT-29 and HCT116 cells (Fig. 4C, D). The results of the transwell assay showed that EpCAM-CAR-T cells could significantly reduce the migration ability of the two cells after co-culture with HT-29 and HCT116 cells (Fig. 4E). The above results showed that EpCAM-CAR-T cells could effectively inhibit EpCAM-positive tumour cells in vitro.

### Cytotoxic effect of epithelial-specific adhesion molecule chimeric antigen receptor T cells on tumour cells in vivo

**Fig. 5:**
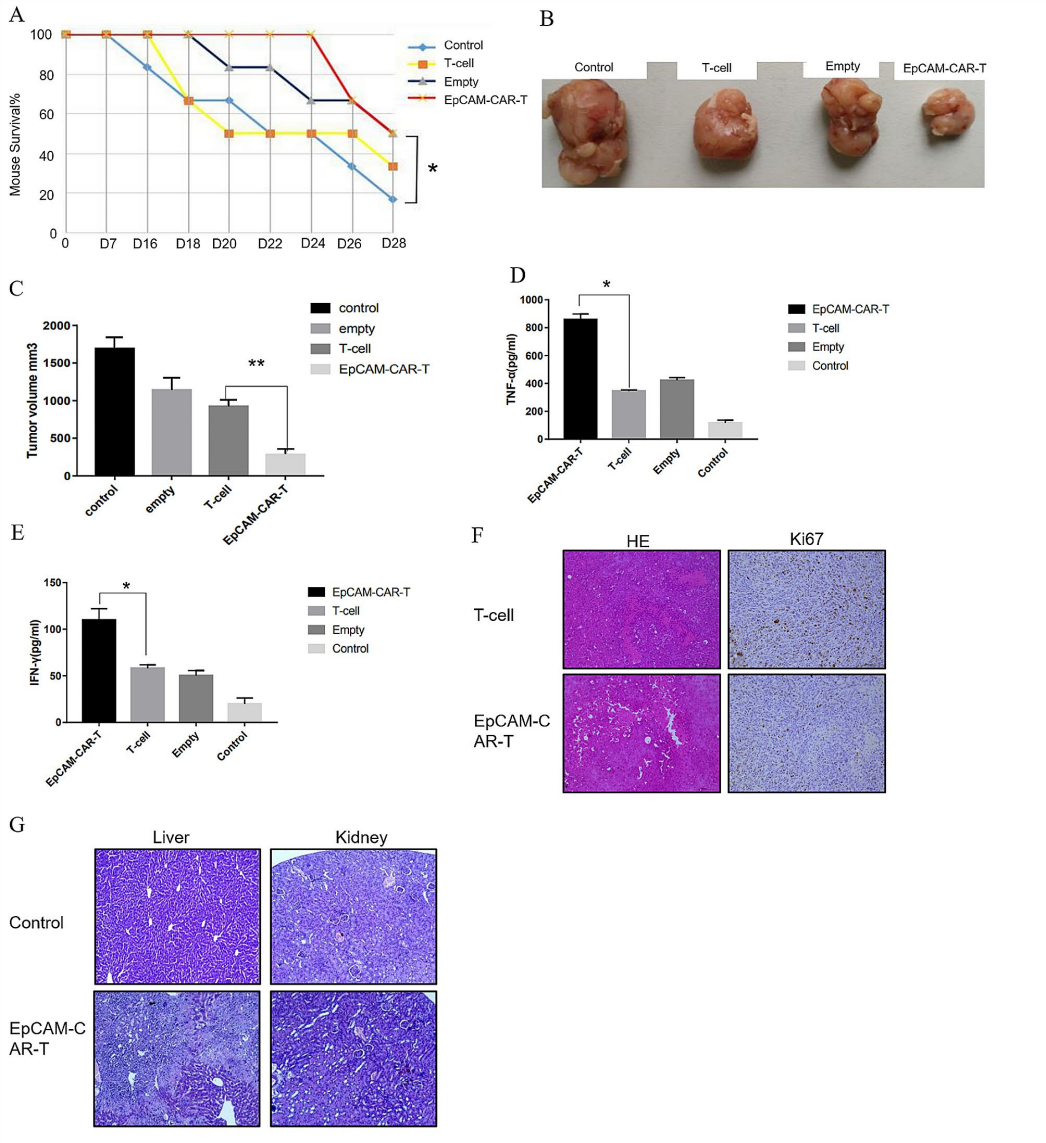
Verification of killing effect of EpCAM-CAR T cells in vivo **A**: Effect of different effector cells on survival in nude mice. Error bars mean ± SD, *: *p* < 0.05, by one-way ANOVA analysis. **B**. The CRC tumors formed by subcutaneous implantations of HT-29 cells. *n* = 6 for each group. **C**. Subcutaneously implanted tumor volume measurements after section. Error bars mean ± SD, **: *p* < 0.01 by two-tailed unpaired student’s t-test analysis. **D**, **E**. ELISA results of TNF-α (D) and INF-γ (E) in serum of nude mice. Error bars mean ± SD, *: *p* < 0.05 by two-tailed unpaired student’s t-test analysis. **F**: Representative images of HE staining as well as Ki67 staining of tumor pathological sections. **G**: Representative pictures of HE staining of liver as well as kidney sections from nude mice

Nude mice with successful tumour formation were randomly divided into four groups, with six mice in each group: the control group (injection medium), the T cell group (injection of common T cells), the empty vector group (injection of T cells transfected with empty vector) and the EpCAM-CAR-T group (injection of T cells transfected with EpCAM-CAR). The experimental results showed that the injection of EpCAM-CAR-T cells significantly improved the survival rate of the nude mice (Fig. 5A). Furthermore, EpCAM-CAR-T cells could also effectively kill tumour cells in vivo and significantly reduce the tumour volume (Fig. 5B, C). The contents of TNF-α and INF-γ in the serum of the nude mice were found to be significantly increased (Fig. 5D, E). Haematoxylin-eosin staining of pathological sections was performed on the tumour, and microscopic findings showed dark purple nuclei, closely arranged cells and occasional secretory cavities of adenocarcinoma, which was consistent with the pathological changes of colorectal adenocarcinoma (Fig. 5F). The Ki67 expression was significantly reduced in tumours injected with EpCAM-CAR-T cells compared with controls. The positive rate of Ki67 was 27% in the T-cell CAM-CAR-T group and 8% in the EpCAM-CAR-T group (Fig. 5F). The liver and kidney of the EpCAM-CAR-T cell-injected group did not change significantly compared with the control group, indicating that EpCAM-CAR-T cells were less cytotoxic (Fig. 5G). The above results showed that EpCAM-CAR T cells also significantly inhibited EpCAM-positive tumour cells in vivo.

## Discussion

The incidence and mortality of colorectal cancer remain high [21]. Colorectal cancer generally occurs in patients over 50 years of age [4] and is often found to be at an advanced stage [22]. Therefore, new treatment methods need to be sought for this cancer. The efficacy of immunotherapy techniques in solid tumours has become the direction of key research. In this study, a third-generation EpCAM-CAR was designed against colorectal cancer-specific antigen EpCAM and successfully constructed by lentiviral infection into T cells, and the effectiveness of this EpCAM-CAR-T cell was verified both in vitro and in vivo.

The occurrence of tumours is closely related to the human immune system. The function of the normal body’s immune system is to recognise mutated foreign cells, perform immune clearance for them and play an immune surveillance role to maintain the balance of the body [23]. Tumour escape pertains to the phenomenon wherein tumour cells evade immune system surveillance and clearance by acquiring functional alterations, disrupting the delicate balance maintained by the immune system [24, 25]. There are many mechanisms of immune escape in tumours, and some are still not well understood [26], but impaired recognition and the presentation of antigens by immune cells is a recognised mechanism [25, 27]. In response to the immune escape mechanism characterised by disrupted immune recognition, diminished or absent T cell responsiveness, and reduced tumour antigen expression in tumour cells, scientists have devised immune cell therapy [8]. The specific execution of this process entails genetically modifying immune cells to express the scFv derived from an antibody specific to a tumour-associated antigen on their cell surface. This results in the presence of a receptor capable of recognising the antigen on the immune cell surface. After the antigen is recognised, it can transmit the signal to the activation signal sequence in the intracellular region and up-regulate the expression of related genes [9, 10]. At present, the relatively mature technology applied is CAR-T cell technology. It has now progressed to the fourth generation [28], and the first generation of CAR-T cells consists of an extracellular scFv sequence and an intracellular CD3ξ signal sequence, of which the scFv sequence is responsible for recognising tumour surface antigens, and the immunoreceptor tyrosine activation motif (CD3ξ) produces signals that up-regulate gene expression, but the first generation is not very effective [29]. Costimulatory molecule signalling molecules have been introduced in both the second- and third-generation CARs [30]. The difference is that the number of costimulatory signalling molecules introduced in the third generation is more than that in the second generation, and costimulatory molecule signals can not only improve the antitumor ability of T cells [31, 32] but also the survival ability of CAR-T cells [33]. These two generations of CAR structures are also the most used and most effective structures at present [34, 35]. Currently, fourth-generation CARs have been developed, and they introduce an intracellular domain co-expressing small molecules (e.g. the proinflammatory cytokine, IL-12) that can trigger cytokine-induced signalling or block some signalling pathways that affect CAR-T cell function to harvest stronger efficacy [36].

Although CAR-T has performed well in the treatment of hematologic malignancies and clinical trials have achieved complete remission [37–41], researchers have found that the clinical effect of CAR-T in the treatment of solid tumours is far from that of hematologic tumours [42, 43]. The reason for this may be that there are many differences between the microenvironment of solid tumours and the hematologic system. The process of effector T cell homing to reach the lesion is also more difficult than that of hematologic tumours. Moreover, solid tumours are different from hematologic tumours, and the heterogeneity of solid tumours determines that targeting an antigen does not kill all solid tumour cells [44, 45]. In addition, unlike haematological tumours, solid tumours have spatial structures and some hindering effects on CAR-T cell infiltration. Additionally, CAR-T cells enter the interior of the tumour with difficulty, resulting in limited killing of solid tumours by these cells. In the treatment of solid tumours, CAR-T cells are depleted when killing tumour cells, whereas in the treatment of haematological tumours, CAR-T cells undergo little depletion [44, 45]. Although scientists have tried multiple drug combinations of CAR-T treatments, the effects of these treatment combinations are always limited and far inferior to their therapeutic effects on haematologic tumours [46]. Therefore, the therapeutic effect of CAR-T on solid tumours needs to be improved.

This study selected EpCAM as a target to design a third-generation EpCAM-CAR. Moreover, EpCAM-CAR-T cells were used to kill EpCAM-positive and negative tumour cells in vitro. Compared with the common T cell group, EpCAM-CAR-T cells had strong a cytotoxic impact against EpCAM-positive tumour cells, and EpCAM-CAR-T cells could secrete a large amount of TNF-α and INF-γ. Therefore, it is speculated that the tumoricidal effect of EpCAM-CAR-T cells constructed in this experiment may be partly related to their massive secretion of TNF-α and INF-γ after activation. Furthermore, TNF-α and INF-γ exerted an adjuvant antitumor effect, allowing EpCAM-CAR-T cells to exert a better antitumor effect. In-vivo experiments fully demonstrated this, and the experimental results revealed that the blank group showed mortality from day 16, and the control group showed extreme abdominal distension in dead nude mice on day 18, while the EpCAM-CAR-T group did not show mortality until day 26, with little abdominal distension. The average tumour volume of each group calculated via the drainage method was found to be smaller in the experimental group than in both the empty vector and control groups (*P* < 0.05). In addition, the mortality rate of the empty vector group was higher than that of the experimental group (*P* < 0.05). The secretion of the serum killer cytokines, TNF-α and IFN-γ, in tumour-bearing nude mice in the experimental group was higher than that in the empty vector and control groups (*P* < 0.05). Tumour sections showed no significant lymphocyte infiltration in each group, which indicated better targeting of EpCAM-CAR-T cells. Pathological section observation of the liver and kidney showed that the liver and kidney tissues of the empty vector group, the control group and the experimental group were basically intact, and no obvious metastatic carcinoma occurred. The above results indicated that EpCAM-CAR-T cells are less cytotoxic.

## Conclusion

In this study, we successfully constructed a third-generation EpCAM-CAR. T cells transfected with EpCAM-CAR demonstrated the ability to effectively target and recognise EpCAM-positive tumour cells. These modified T cells exhibited enhanced functionality, secreting a substantial amount of killer cytokines, including TNF-α and IFN-γ. In-vivo experiments further revealed their superior capacity to inhibit the growth of colorectal cancer, leading to an improved quality of life for tumour-bearing nude mice. However, while EpCAM-CAR plays a therapeutic role, it may also produce off-target effects and cause a cytokine storm; therefore, further studies on the mechanism of action of EpCAM-CAR-T cells and the causes of related side effects are needed. Off-target effects can also be reduced using Dual CARs/Tandem CARs/synNotch CARs.

### Electronic supplementary material

Below is the link to the electronic supplementary material.

Supplementary Material 1

## Data Availability

All data generated or analyzed during this study are included in this published article.
