# Supplementary material for: Chimeric antigen receptor-T cells targeting epithelial cell adhesion molecule antigens are effective in the treatment of colorectal cancer
Source: BMC Gastroenterol. 2024 Aug 6;24:249. doi: 10.1186/s12876-024-03286-9 (PMC11302356; doi:10.1186/s12876-024-03286-9)

Figure 2F EpCAM


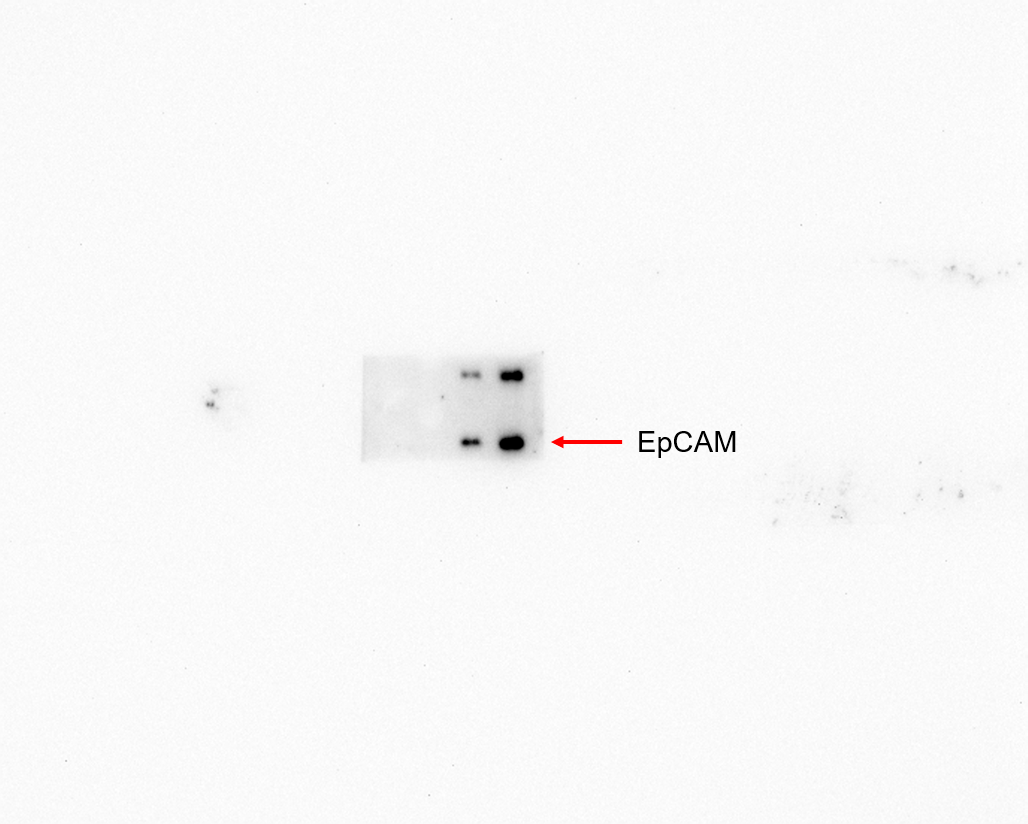


EpCAM Original figure


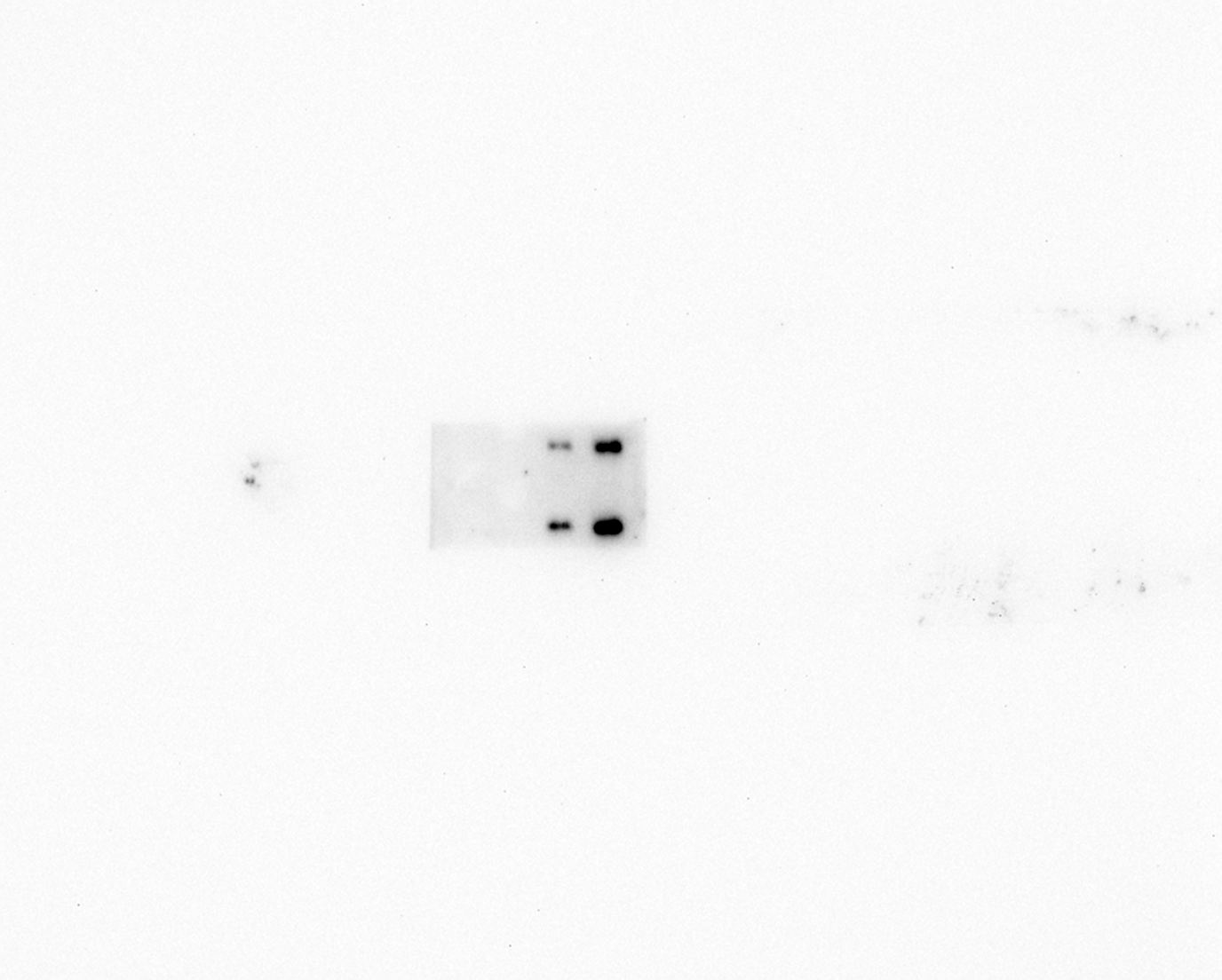


EpCAM blank Original figure


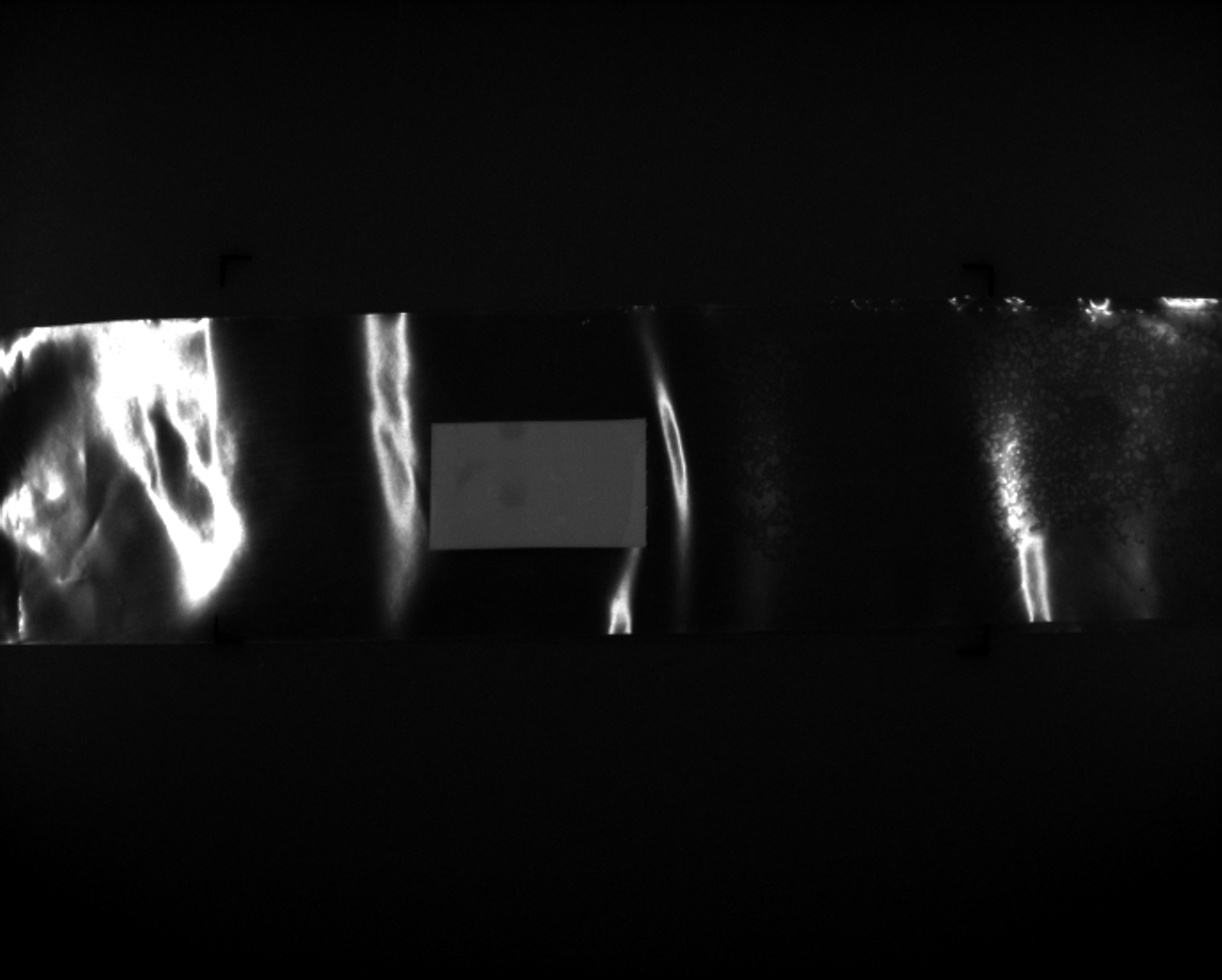


Figure 2F GAPDH


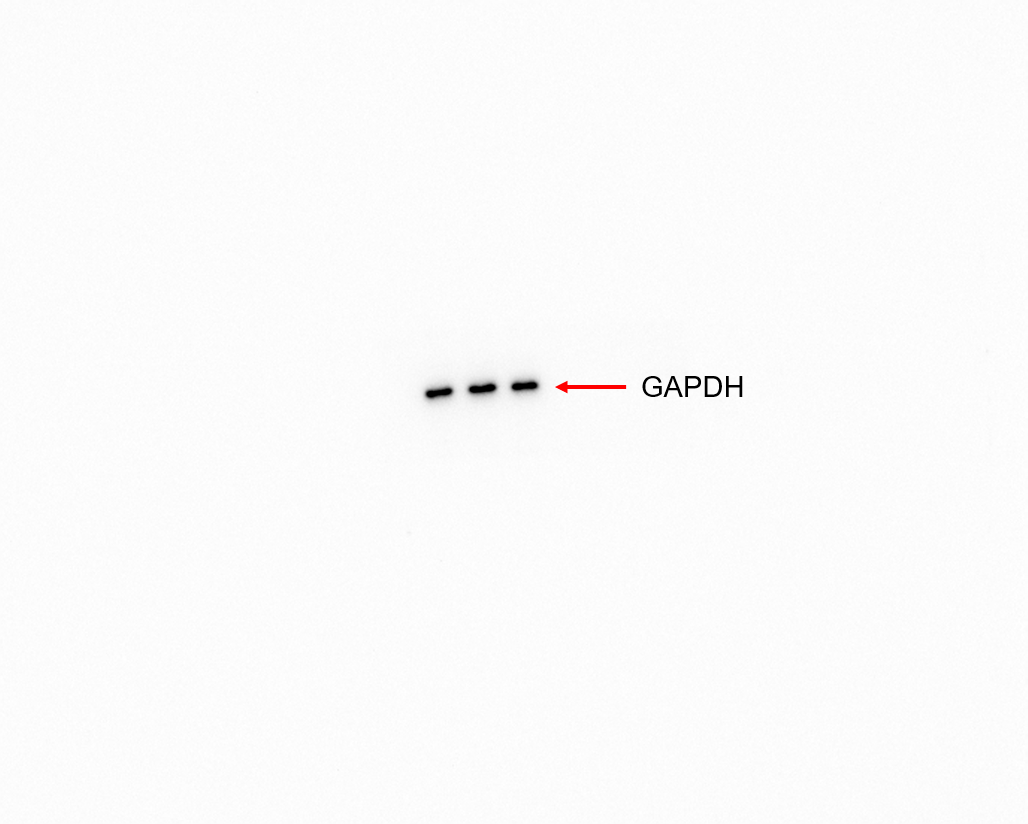


GAPDH Original figure


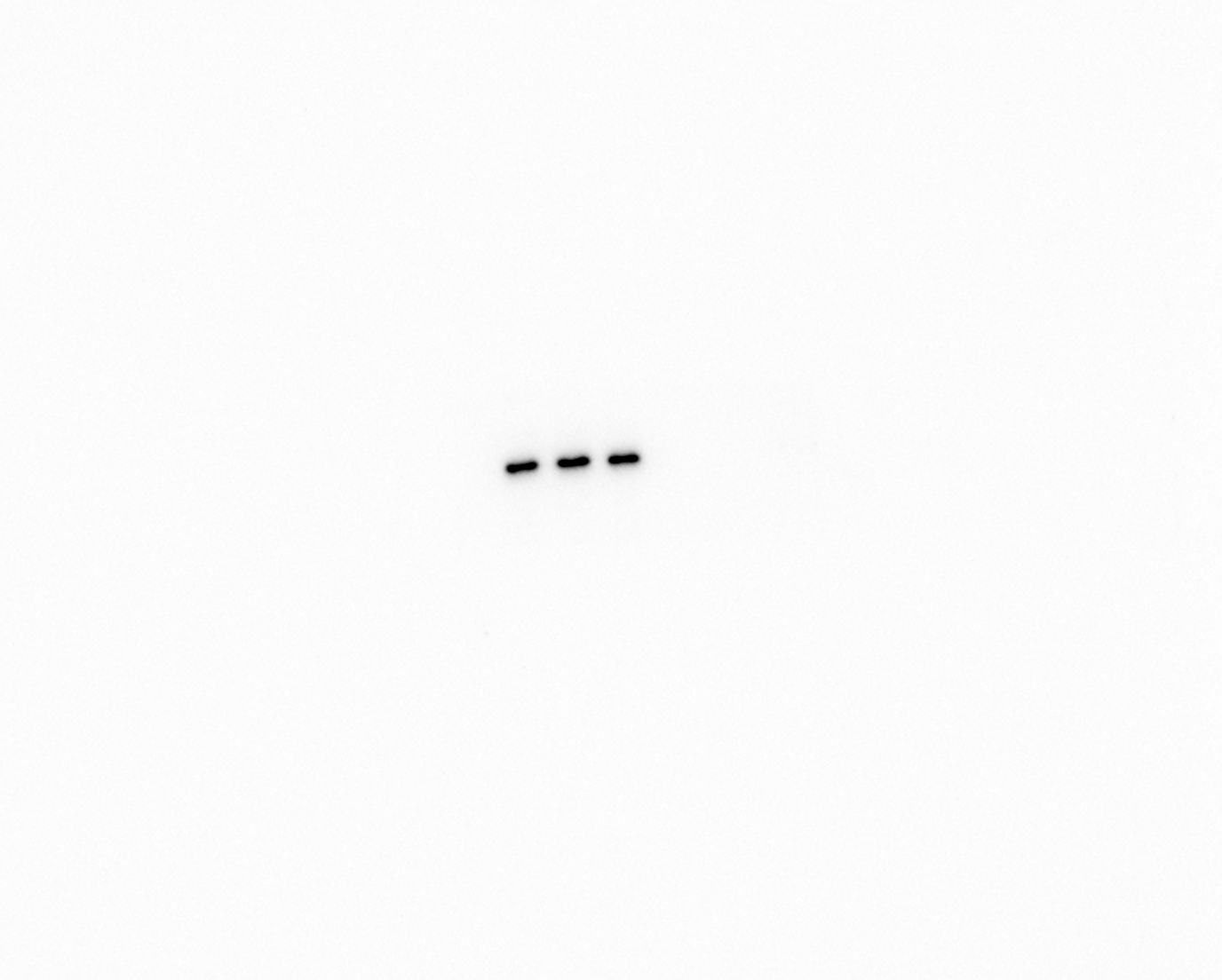


GAPDH blank Original figure


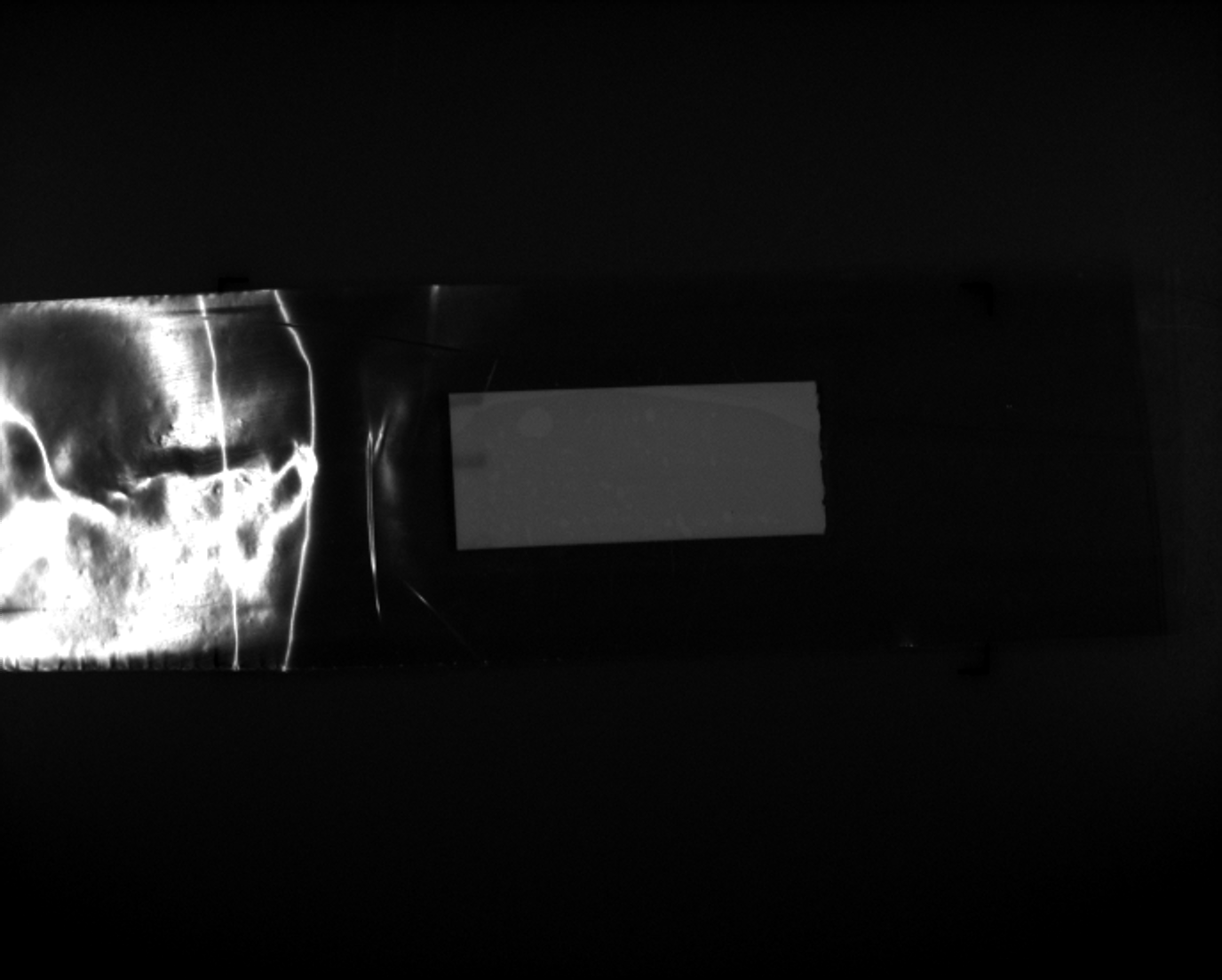

Supplement: Supplementary file 1 — Supplementary Material 1 [file 12876_2024_3286_MOESM1_ESM.docx]
